# Thoracic Endovascular Aortic Repair for Ruptured Intercostal Patch Aneurysm Following Thoracoabdominal Aortic Aneurysm Repair

**DOI:** 10.3400/avd.cr.20-00053

**Published:** 2020-09-25

**Authors:** Soichiro Henmi, Hidekazu Nakai, Katsuhiro Yamanaka, Atsushi Omura, Takeshi Inoue, Kenji Okada

**Affiliations:** 1 Division of Cardiovascular Surgery, Department of Surgery, Kobe University Graduate School of Medicine

**Keywords:** thoracoabdominal aortic aneurysm, intercostal patch aneurysm, thoracic endovascular aortic repair

## Abstract

Aneurysmal change of reconstructed intercostal arteries is believed to be a rare complication after thoracoabdominal aortic repair. To our knowledge, there is no guideline or randomized controlled trials regarding intercostal patch aneurysm management. Therefore, the optimal treatment is still controversial. We describe a successful case of emergent thoracic endovascular aortic repair for ruptured intercostal patch aneurysm in an 83-year-old man following thoracoabdominal aortic aneurysm repair. Our experience illustrated that gradual expansion of large blocks of aortic wall reconstruction should be closely monitored after primary thoracoabdominal aortic replacement.

## Introduction

Spinal cord injury (SCI) remains one of the most serious complications of thoracoabdominal aortic aneurysm repair, and a reliable method to prevent this has not yet been established. We believe that reattachment of the responsible intercostal arteries (ICAs) is considered one of the most necessary adjuncts to avoid SCI. However, some of these patients required reoperation for patch aneurysms in the segmental arteries after primary thoracoabdominal aortic repair. In this literature, we describe a successful case of emergent thoracic endovascular aortic repair (TEVAR) for ruptured intercostal patch aneurysm in an 83-year-old man following thoracoabdominal aortic aneurysm repair.

## Case Report

**Figure 1:**
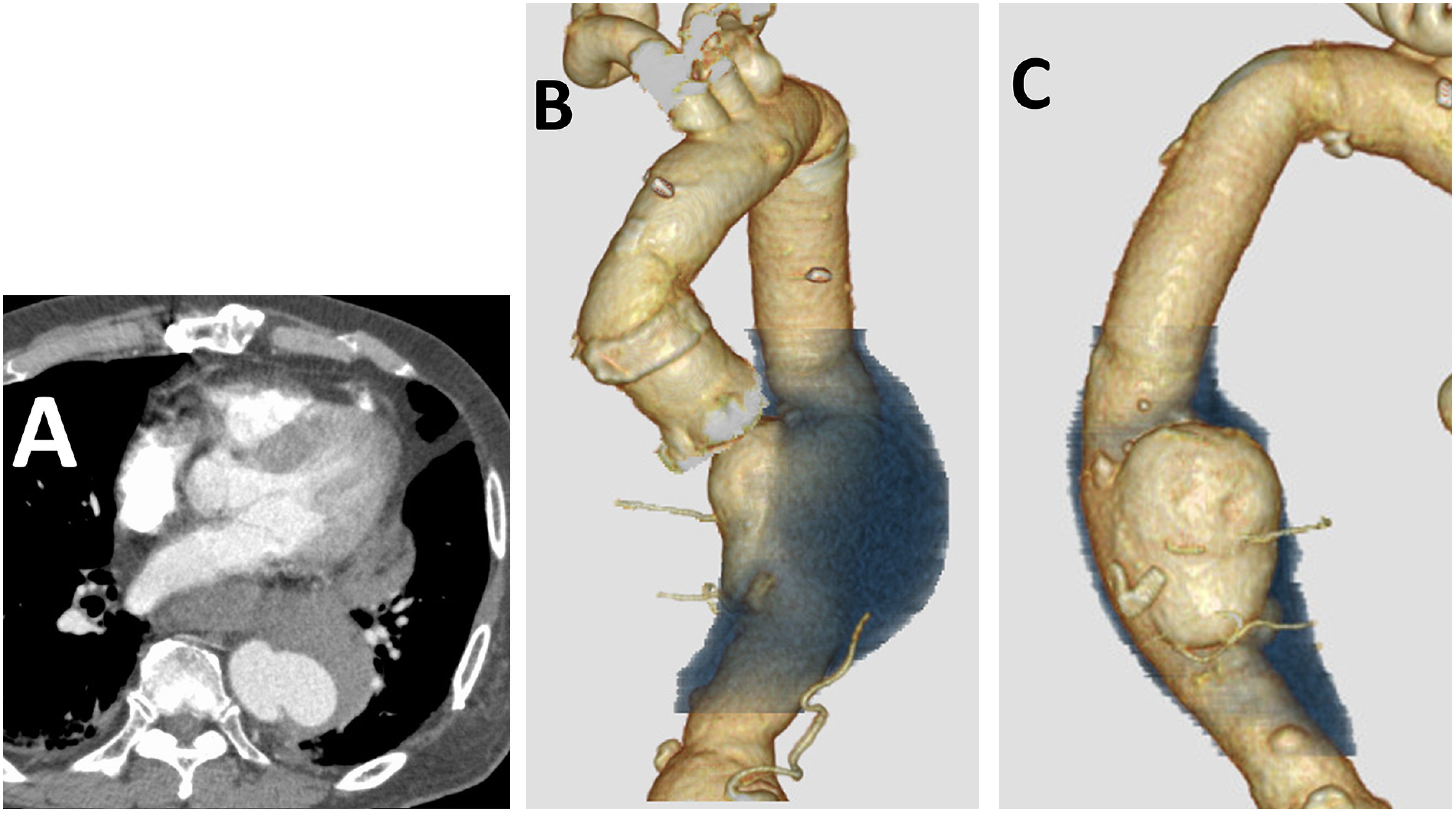
Fig. 1 Preoperative enhanced computed tomography scan showing an intercostal patch aneurysm measuring 64×60×61 mm with rupture and a huge hematoma. (**A**): Axial view; (**B**), (**C**): 3-dimensional view.

**Figure 2:**
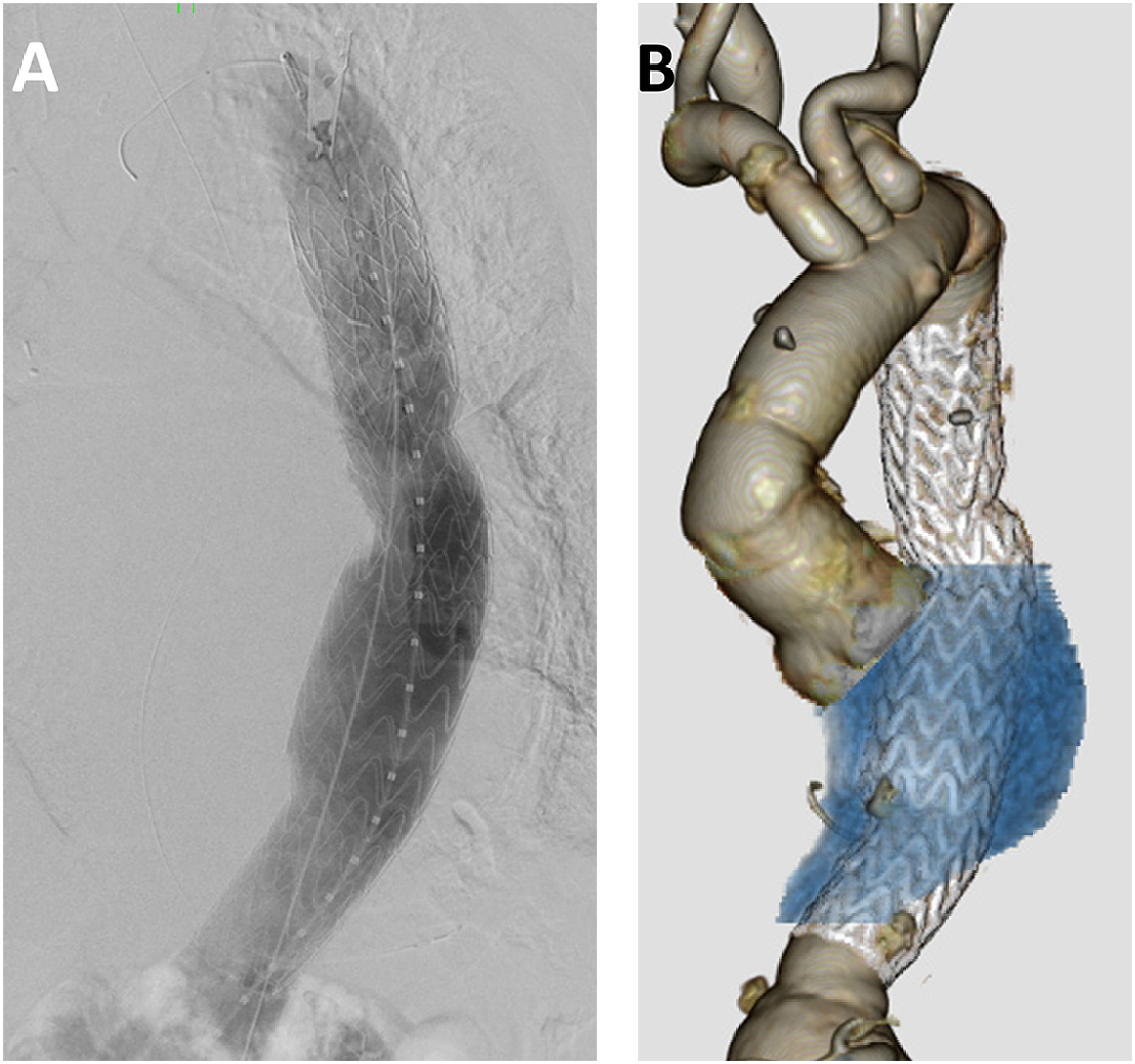
Fig. 2 Postoperative digital subtraction angiography (**A**) and enhanced computed tomography scan (**B**) were showing complete sealing of the intercostal patch aneurysm with stent graft of thoracic endovascular aortic repair and marked shrinking without endoleaks.

**Figure 3:**
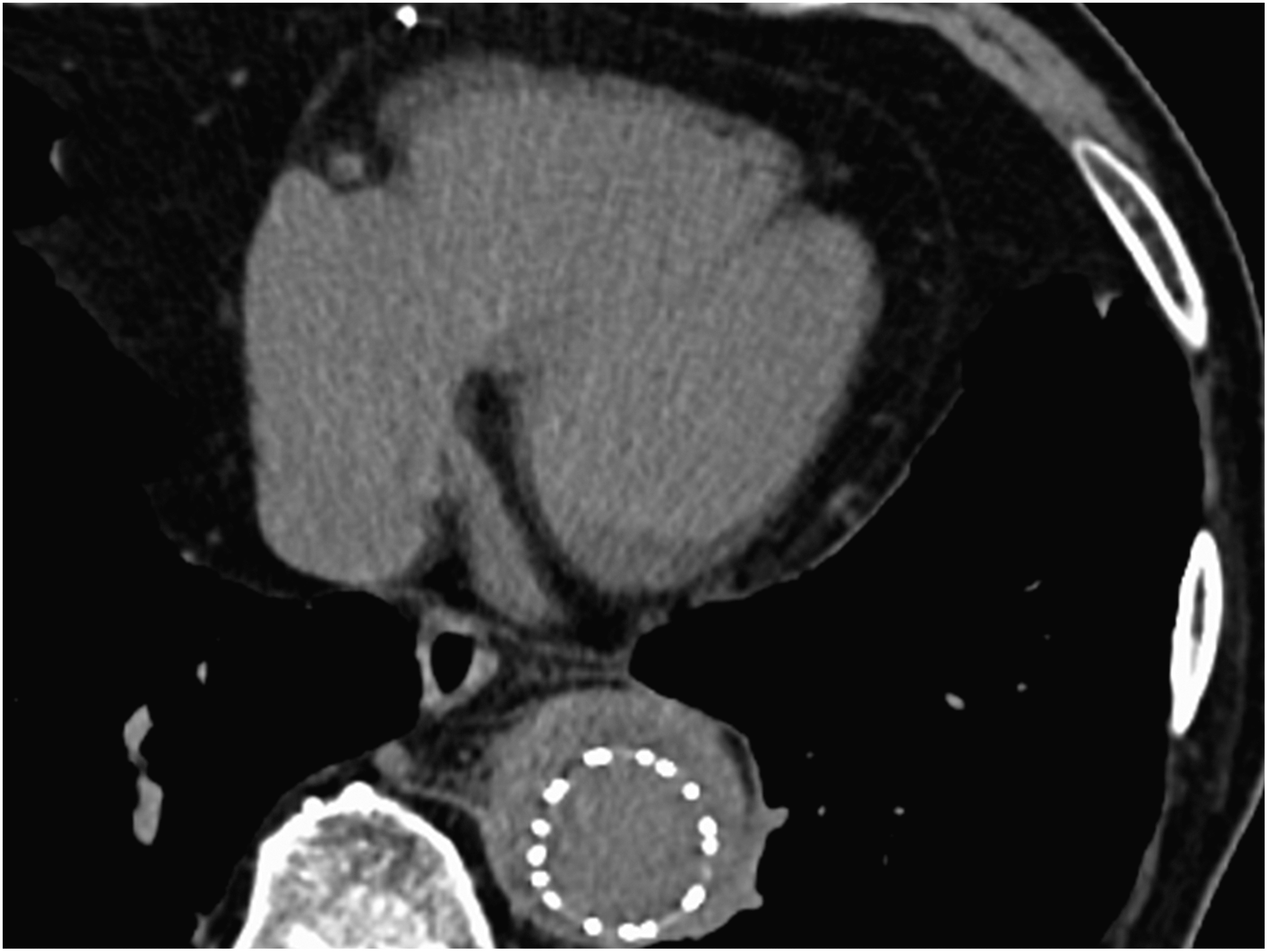
Fig. 3 Non-enhanced computed tomography scan 1 year after surgery was showing significant shrinking of intercostal patch aneurysm without hematoma.

An 83-year-old man with sudden onset of back pain was referred to our hospital. A type B aortic dissection had been diagnosed at 62 years of age. He had previously undergone three aortic operations: descending thoracic aortic replacement, thoracoabdominal aortic replacement, and total arch replacement. Thoracoabdominal aortic replacement had been performed at the age of 71 with a 22-mm woven Dacron graft from the preexisting proximal descending thoracic aortic graft to the supra celiac artery. The Adamkiewicz artery was identified to be the left thoracic (Th) ninth ICA by preoperative enhanced computed tomography (CT) scan. During this surgery, the patent orifices of Th8-10 ICAs were anastomosed to a large side hole on the graft using an island technique. Postoperatively, the annual follow-up CT revealed that the patch of reconstructed Th8-10 ICAs remained patent and the residual aortic wall had gradually expanded. However, he did not wish a re-intervention for intercostal patch aneurysm because he was asymptomatic. An enhanced CT showed that the patch of the reconstructed Th8-10 ICAs had increased to 64×60×61 mm in diameter and ruptured with a huge hematoma (**Fig. 1**) 12 years after this operation. Emergent TEVAR was done under general anesthesia and monitoring with digital subtraction angiography. The right common femoral artery was exposed, and 50 U/kg intravenous bolus of heparin was given at the beginning of TEVAR. A 0.035-inch Radifocus guidewire (TERUMO Co., Tokyo, Japan) was inserted through the right common femoral artery, and an 8-French sheath was introduced over the guidewire. The Conformable GORE TAG (CTA G) device (W. L. Gore & Associates, Flagstaff, AZ, USA) sized 31×20 cm was delivered and deployed to seal the intercostal patch aneurysm (**Fig. 2**). Both the proximal and distal landing sites of the CTA G were at the preexisting 22-mm woven Dacron graft site with 4 cm of landing zone. No endoleak was detected on digital subtraction angiography and enhanced CT scan. The patient’s postoperative course was uneventful, without any SCI, and he was discharged in good condition at 14 days after his operation. One year after surgery, CT revealed that the aneurysm was shrinking markedly without hematoma (**Fig. 3**).

## Discussion

Aneurysmal change of reconstructed ICAs is believed to be a rare complication after thoracoabdominal aortic repair. Kulik and colleagues^1)^ reported that 11 of 155 cases (7.1%) developed patch aneurysms 64 months after the initial surgery, of which 8 underwent repeat open operation and 3 underwent endovascular stent graft replacement. Juthier and colleagues^2)^ reported four cases with endovascular exclusion of patch aneurysms in ICAs 70 months after thoracoabdominal aortic surgery. There were no inhospital deaths or postoperative SCI in both reports. In a previous report, 79 months after thoracoabdominal aortic surgery, four patients required redo open operation for ICA patch aneurysm.^3)^ Based on these reports, we determined that an intercostal patch aneurysm may not be a rare complication.

To our knowledge, the mode of surgery for an ICA patch aneurysm is either redo open surgery or endovascular repair. Open operation can completely exclude the aneurysm and re-reconstruct ICAs. In contrast, re-thoracotomy has the risk of more bleeding or lung injury, and prolonged hemodynamic instability might cause SCI. TEVAR has a potential risk of endoleak and can lead to sacrifice of all patent ICAs. However, the landing zones are located inside the previous graft, thus limiting the risk of type I endoleaks in such a scenario.

We believe that ICA reconstruction method is also an important factor in determining whether the intercostal patch will lead to an aneurysm or not. Kulik and colleagues^1)^ reimplanted patent multiple ICAs with a Carrel patch, which was similar to island fashion. Kitahara and colleagues^4)^ reported a case of hybrid TEVAR for an intercostal patch aneurysm, which included reimplantion by the island technique. In a previous report, the island technique was also performed in all five patients with intercostal patch aneurysm. Our findings suggest that the method of island reconstruction might be a risk factor for developing an aneurysm. Island reconstruction should be avoided, especially in patients with Marfan syndrome or non-elderly patients.

SCI remains one of the most serious complications after treating intercostal patch aneurysm. In this case, we could not use motor-evoked potential or cerebrospinal fluid drainage because the patient had hemodynamic instability. We had to cover the artery of Adamkiewicz for completely sealing ruptured patch aneurysm. In this situation, we tried to maintain high blood pressure for using vasopressor soon after stent graft was deployed. If SCI occurred postoperatively, we made the decision to insert the cerebrospinal fluid drainage immediately. Fortunately, there was no physical finding of SCI after TEVAR.

In our case, the intercostal patch aneurysm was ruptured. To the best of our knowledge, no cases of ruptured intercostal aneurysms except for pseudoaneurysm have been found in previous literature. Our experience illustrated that gradual expansion of large blocks of aortic wall reconstruction should be closely monitored after primary thoracoabdominal aortic replacement.

## Conclusion

TEVAR for ruptured intercostal patch aneurysm after thoracoabdominal aortic replacement was successfully performed.

## Additional Note

We have obtained written informed consent from the patient for reporting this case.
